# Commentary: Physical Functional Capacity and C-Reactive Protein in Schizophrenia

**DOI:** 10.3389/fpsyt.2018.00007

**Published:** 2018-01-26

**Authors:** Ruth Elliesen, Andreas Walther

**Affiliations:** ^1^Biological Psychology, TU Dresden, Dresden, Germany

**Keywords:** schizophrenia, C-reactive protein, inflammation, physical functioning, antipsychotics

## Introduction

Schizophrenia has recently been related to reduced physical functioning (1, 2). The association between reduced physical exercise, worse body composition, and schizophrenia is already well established (3, 4). Furthermore, it has been shown that physical exercise reduces positive as well as negative symptoms and increases the quality of life of patients with schizophrenia (5). It also improves neurocognitive functions such as reaction time, cognitive flexibility, and executive function (6). These results refer to moderate rather than exhausting physical activity (5, 6). Physical exercise is also related to lower inflammation markers (7), which is interesting as schizophrenia shows an association with inflammatory dysregulation. Studies show elevated cytokine (8, 9) and C-reactive protein (CRP) levels, indicating inflammatory markers as potential biomarkers for schizophrenia (10). Interestingly, elevated CRP levels in pregnant women were associated with a higher risk of the offspring developing schizophrenia (11). Furthermore, CRP levels show a high heritability (12), which may be important in the context of disease establishment and progression.

Atypical and typical antipsychotics seem to have different effects with regard to the inflammatory state. Individuals taking atypical antipsychotics such as quetiapine and olanzapine show higher levels of CRP compared with other antipsychotics (13). Clozapine treatment (another atypical antipsychotic) after a first episode was accompanied by increased CRP levels of about 600% compared with baseline (14). The increased CRP levels might reflect a state marker of an acute illness phase as no elevated CRP levels were identified in the follow-up measurement 1 year later (14). However, antipsychotics in general are thought to have anti-inflammatory effects, reducing CRP levels or the pro-inflammatory interleukin (IL)-6 (15), and simultaneously increasing the level of anti-inflammatory markers such as IL-4 and IL-10 (16). Therefore, standardization of antipsychotic medication is required when investigating the effects of schizophrenia on the immune system, since different antipsychotics affect the immune system in different ways (15).

Besides its potential function as a biomarker for schizophrenia, CRP is also synthesized by hepatocytes under the regulatory control of IL-6, released by macrophages and adipocytes (17). Elevated IL-6 levels have also been linked to schizophrenia (8) and were especially related to negative symptoms (18). Furthermore, IL-6 was shown to be increased prior to the onset of schizophrenia (18).

Therefore, inflammatory processes seem to play a crucial role in the pathophysiology of schizophrenia, while physical functioning is further associated with this illness. Thus, a dynamically interacting network of these three components emerges, and the study by Szortyka and colleagues investigated the association of schizophrenia, inflammatory markers, and physical functional capacity.

## Measures and Approaches

For this purpose, a cross-sectional study was designed, including stable outpatients with a diagnosis of schizophrenia. Forty patients aged between 18 and 60 took part in this study, whereby 18% of the participants were female and the remaining 82% male. All patients already had a history of schizophrenia with at least four episodes, and all patients were receiving psychopharmacological treatment. Most of the patients (*N* = 28) had been suffering from schizophrenia for more than 7 years. To test the physical functional capacity, the participants completed the 6-minute-walk test (6MWT) and blood pressure, heart rate (HR), respiratory rate, peripheral oxygen saturation, and dyspnea were measured at the beginning and at the end of the test, while HR and blood oxygen saturation (BOS) were also measured during the test. Blood samples were collected to examine CRP.

## Associations Between Physical Functioning, CRP, and Schizophrenia

The examined physical functioning (e.g., respiratory rate, BOS, blood pressure, and 6MWT) was lower in the patients compared with the average physical functioning of the general population. In addition, the HR and CRP levels were higher in the clinical sample compared with the general population. Only 3 of the total 40 patients reached the minimum predicted distance. The impaired 6MWD (6-minute-walk distance, difference between actual and predicted distance) and dyspnea were correlated to CRP and an association between impaired physical functioning and elevated levels of CRP in patients suffering from schizophrenia emerged.

## Integrating Results and Conclusion

The present study was the first to identify a link between reduced functional capacity and an increased inflammatory marker in patients with schizophrenia. This finding needs to be highlighted since most existing studies have focused on physical exercise to improve functions of schizophrenic patients and not on the “baseline” physical functioning. Still, it remains unclear whether reduced physical functioning is a direct result of schizophrenia or whether it is due to a lack of motivation to exercise because of the different consequences of schizophrenia. Future studies need to assess this issue since it was shown that patients with schizophrenia spend less time doing physical activities, potentially leading to impaired physical functioning (19). Furthermore, the association of schizophrenia and increased inflammatory markers is well established and indicates that schizophrenia is further related to “sickness behavior.” Sickness behavior results from inflammatory cytokines such as IL-1α and β, TNF-α, and IL-6 being released from the activated immune system, which then act on the brain, where further inflammatory cytokines are produced (20). Sickness behavior is generally associated with reduced vigorous and physical activity (20). Therefore, this could also be a moderator in the processes mentioned above and merits further investigation.

There were two subgroups in the study (schizophrenia less or more than 7 years) and as no further information was provided about the differences between these groups, they probably did not differ in the physical tests. Nevertheless, it would have been relevant to clarify that and to further elucidate if there were differences with regard to gender. Kahl and colleagues showed that reduced muscle mass and depression are associated with male gender and chronicity (3), so it might be probable that there are sex-specific effects in schizophrenia which are related to muscle mass and chronicity as reported for other psychopathologies (21, 22).

The authors failed to provide more information on the association between CRP and physical functioning. However, the measurement of additional inflammatory and anti-inflammatory markers such as IL-6, NF-KB, TNF-alpha, or IL-10 in a longitudinal setting would be relevant to clarify the underlying pathophysiology linking schizophrenia, inflammation, and reduced physical functioning, thereby revealing causal relations. Furthermore, the inflammation regulating role of steroid hormones needs to be taken into account, when examining the effects of inflammatory markers in psychiatric conditions (23–26). The time for big data examining multiple omic layers called trans-omics has come and will provide insight into the pathophysiology of schizophrenia and many other psychiatric conditions on a new level (27, 28).

The authors furthermore refer to the state of the patients as “stable schizophrenia” (1), indicating that they are in the residual phase with no symptoms at the time of the examination or predominantly negative symptoms. However, it would have been relevant to mention that since it has been shown that CRP levels in patients with a bipolar disorder differ depending on the disease state (manic, depressed, remitted), with the highest levels occurring during mania and the lowest levels emerging during remission, yet still being higher than healthy controls (29). Therefore, there might be a relation between the CRP level and different disease states in schizophrenia as well.

Moreover, the study did not include a control group with healthy individuals, which would have significantly strengthened the identified differences. Importantly, a lack of information is present with regard to the specific medication the patients took. This information should be provided in future studies in order to differentiate between the types of antipsychotics and their effects on the disease, inflammatory state, and physical functioning to a better extent.

In conclusion, the study of Szortyka and colleagues provides new and promising insights in the field of biomarker research for schizophrenia and the relation to physical functioning. Future studies should include a broad spectrum of relevant cytokines to present the complex picture of interacting cytokines in schizophrenia, clinical tests (e.g., the PANSS), and more specific information about the patients and their medication. The pathophysiology of schizophrenia includes a network of functions and markers varying in patients suffering from schizophrenia compared with the general population. To elucidate this complex interacting system, large studies are required to capture probable moderation or mediation processes. This would help to gain a better understanding of the underlying pathophysiology of schizophrenia and ultimately develop more efficient therapies.

## Author Contributions

RE wrote the first draft of this manuscript and edited subsequent versions. AW revised the first draft and edited subsequent versions.

## Conflict of Interest Statement

The authors declare that the research was conducted in the absence of any commercial or financial relationships that could be construed as a potential conflict of interest.
